# Mitochondrial function in individuals at clinical high risk for psychosis

**DOI:** 10.1038/s41598-018-24355-6

**Published:** 2018-04-18

**Authors:** Tania Da Silva, Abbie Wu, Isabelle Laksono, Ivana Prce, Margaret Maheandiran, Michael Kiang, Ana C. Andreazza, Romina Mizrahi

**Affiliations:** 1 Research Imaging Centre, Centre for Addiction and Mental Health, Toronto, Ontario Canada; 2 Department of Pharmacology & Toxicology, University of Toronto, Toronto, Ontario Canada; 3 Institute of Medical Science, University of Toronto, Toronto, Ontario Canada; 4 Department of Psychiatry, University of Toronto, Toronto, Ontario Canada; 5 Campbell Family Mental Health Research Institute, Centre for Addiction and Mental Health, Toronto, Ontario Canada

## Abstract

Alterations in mitochondrial function have been implicated in the etiology of schizophrenia. Most studies have investigated alterations in mitochondrial function in patients in which the disorder is already established; however, whether mitochondrial dysfunction predates the onset of psychosis remains unknown. We measured peripheral mitochondrial complex (I–V) function and lactate/pyruvate levels in 27 antipsychotic-naïve individuals at clinical high risk for psychosis (CHR) and 16 healthy controls. We also explored the association between mitochondrial function and brain microglial activation and glutathione levels using a translocator protein 18 kDa [^18^F]FEPPA PET scan and ^1^H-MRS scan, respectively. There were no significant differences in mitochondrial complex function and lactate/pyruvate levels between CHR and healthy controls. In the CHR group, mitochondrial complex III function (r = −0.51, *p* = 0.008) and lactate levels (r = 0.61, *p* = 0.004) were associated with prodromal negative symptoms. As previously reported, there were no significant differences in microglial activation and glutathione levels between groups, however, mitochondrial complex IV function was inversely related to microglial activation in the hippocampus in CHR (r = −0.42, *p* = 0.04), but not in healthy controls. In conclusion, alterations in mitochondrial function are not yet evident in CHR, but may relate to the severity of prodromal symptoms, particularly negative symptoms.

## Introduction

Altered brain energy metabolism and mitochondrial dysfunction have been implicated in the etiology of schizophrenia^1^. Mitochondria generate cellular energy in the form of ATP through the electron transport chain (ETC)^2^. The ETC consists of five multi-subunit protein complexes, complexes I, II, III, IV and V, located on the inner mitochondrial membrane. Mitochondria play an important role in regulating cellular bioenergetics including redox signaling, calcium homeostasis, and apoptotic cell death^2^. Thus, compromised mitochondrial function can result in impaired calcium buffering, apoptosis and over-production of reactive oxygen species (ROS)^3^. In addition to their role in energy production, mitochondria are also involved in regulating neuronal development and synaptic plasticity^3^. As such, mitochondrial dysfunction may alter critical neuronal processes underlying abnormal brain development and cognitive impairment in psychosis.

Alterations in mitochondrial function in schizophrenia are supported by converging evidence from genetic, post-mortem, peripheral and imaging studies^3,4^. Genetic studies have identified single-nucleotide polymorphisms (SNPs) in mitochondrial DNA (mtDNA) and mitochondrial-related genes as risk factors for schizophrenia^5–8^. In addition, several post-mortem studies have reported reductions in the expression of mitochondrial-related genes^9,10^, particularly genes encoding mitochondrial complexes^1,11,12^, as well as reduced enzymatic activity in multiple brain regions^13^, although others have failed to replicate these findings^14^. Post-mortem studies are confounded by numerous factors including cause of death, duration of illness and long-term medication use. In order to avoid the limitations associated with post-mortem studies, mitochondrial complex function has been measured in peripheral tissues in living patients.

Alterations in mitochondrial complex activity have been consistently reported in blood cells of schizophrenia patients. Reduced mitochondrial complex I activity has been reported in lymphocytes and platelets of SCZ patients chronically treated with antipsychotics compared to healthy controls^15,16^, with no differences in complex II and III activity^17^. Conversely, increased complex I activity was observed in platelets of medicated and unmedicated schizophrenia patients in acute exacerbation^18,19^. In addition, studies have also reported mitochondrial-induced impairments in brain energy metabolism^20^. Phosphorous magnetic resonance studies (^31^P-MRS) reported lower levels of ATP and phosphocreatine (PCr) in brain of patients with schizophrenia^21,22^. Additionally, elevated lactate^1,23–25^ and pyruvate levels^26^ have also been reported in schizophrenia patients, consistent with mitochondrial dysfunction and a shift toward anaerobic metabolism.

Mitochondrial dysfunction and immune activation are tightly linked; mitochondrial dysfunction can modulate inflammatory signaling pathways whereas immune processes can also alter mitochondrial function^27^. Mitochondria are a major source of ROS^28^. During normal mitochondrial metabolism, only a small proportion of electrons escape the ETC resulting in the formation of the ROS superoxide anion (O_2_^−^)^29,30^. Mitochondrial O_2_^−^ is converted to hydrogen peroxide and in the presence of reduced metal ions can form highly reactive hydroxyl radicals^31^. These ROS are readily reduced by controlled antioxidant defense mechanisms, such as glutathione, superoxide dismutase, and glutathione peroxidase, thus maintaining redox balance. Impaired mitochondrial function often produces an excess of ROS and depletion of antioxidants resulting in oxidative stress^32,33^; however, a recent proton magnetic resonance spectroscopy (^1^H-MRS) study reported no alterations in glutathione levels, the major brain antioxidant, between CHR and healthy controls^34^. Oxidative stress can cause further damage to cellular proteins, lipids and nucleic acids, including mitochondrial DNA^35,36^. In addition, recent studies have suggested a role of mitochondria as potent activators of the inflammatory response^27^. ROS produced by mitochondria serve as critical signaling molecules in the activation of redox-sensitive inflammatory pathways^27,37^. ROS are also generated by activated glial cells including microglia and macrophages. Mitochondria are particularly vulnerable to oxidative damage induced by activated inflammatory cells. Thus, mitochondrial dysfunction could lead to alterations in redox balance and immune activation, further increasing mitochondrial damage and oxidative stress; however, the interplay between mitochondrial function, microglial activation (indexed by translocator protein 18 kDa [^18^F]FEPPA positron emission tomography, PET) and redox regulation (indexed by ^1^H-MRS glutathione) has never been investigated *in vivo* in human brain. Further, most studies have investigated alterations in mitochondrial function in patients in which the disorder is already established, however, whether mitochondrial dysfunction is present before the onset of psychosis remains unknown.

In the current study, we tested for the first time alterations in (i) peripheral mitochondrial complex function and (ii) peripheral lactate and pyruvate levels in antipsychotic naïve CHR individuals compared to healthy controls. In addition, we also explored the association between mitochondrial function and brain microglial activation and glutathione levels.

## Results

Demographic and clinical information is shown in Table 1. One CHR was removed from the mitochondrial complex function analysis due to unreliable values. The groups did not differ according to gender, age, BMI or NNT. However, there were more tobacco users in the CHR group as compared to healthy controls (*p* = 0.03). Most of the participants in the CHR group were antipsychotic naive (n = 22). Five CHR individuals were currently on low-dose antipsychotic treatment with either Risperidone (one with 0.5 mg and two with 1 mg), Quetiapine (75 mg) or Aripiprazole (5 mg). All participants had a negative urine drug screen, except for two CHR who had a positive drug screen for cannabis. There were no significant differences in mitochondrial complex (I-V) function and lactate and pyruvate levels between CHR and healthy controls. In the CHR group, mitochondrial complex III function and lactate levels were associated with prodromal symptom severity, particularly negative symptoms. As previously reported, no significant differences in microglial activation and glutathione levels were observed between groups. However, in CHR, mitochondrial complex IV function was inversely related to microglial activation in the hippocampus, but not in healthy controls.

**Table 1:** Demographic and clinical information.

Demographics		Healthy controls (n = 16)	Clinical high risk (n = 27)		
Age (years), SD		21.25 ± 2.05	20.26 ± 1.72	*t* = 1.70	*p* = 0.10
Gender	Male	5	15	χ^2^ = 2.39	*p* = 0.12
	Female	11	12		
BMI, SD		23.99 ± 5.00	23.94 ± 5.66	*U* = 212	*p* = 0.92
NNT, SD^1^		6643.56 ± 2598.70	6719.50 ± 2560.16	*U* = 207	*p* = 0.98
Drug use (current)^2^	Tobacco	0	7	χ^2^ = 4.96	*p* = 0.03
	Cannabis	0	2	χ^2^ = 1.24	*p* = 0.27
	None	16	20		
Antipsychotic use^3^		0	5		
SOPS, SD	Total		35.70 ± 9.64		
	Positive		11.78 ± 3.38		
	Negative		10.78 ± 5.02		
	Disorganization		3.63 ± 2.31		
	General		8.48 ± 3.79		
RBANS, SD	Total	86.25 ± 13.97	88.89 ± 14.28		
	Immediate memory	94.88 ± 15.58	95.19 ± 15.36		
	Visuospatial memory	81.25 ± 21.20	85.52 ± 12.97		
	Language	84.94 ± 18.21	83.81 ± 21.68		
	Attention	97.94 ± 14.69	99.74 ± 17.03		
	Delayed memory	89.25 ± 13.48	93.81 ± 10.03		

Abbreviations: BMI, body mass index; NNT, nicotinamide nucleotide transhydrogenase; RBANS, Repeatable Battery for the Assessment of Neuropsychological Status; SD, standard deviation; SOPS, scale of psychosis-risk symptoms.

^1^One CHR was excluded from the mitochondrial complex function analysis due to unreliable values.

^2^All participants had a negative urine drug screen for cannabis, ethanol, methadone, and cocaine at baseline except two CHR who had a positive urine drug screen for cannabis.

^3^CHR were currently on antipsychotic treatment with 75 mg of Quetiapine, one with 0.5 mg and two with 1.0 mg of Risperidone and the last one with 5 mg Aripiprazole.

### Peripheral Mitochondrial Complex Function

**Figure 1:**
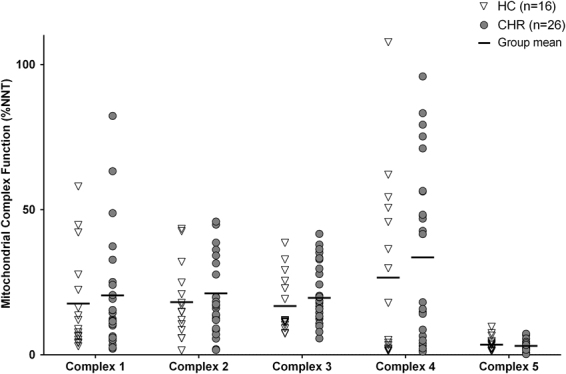
Mitochondrial complex I–V function in clinical high risk (CHR) for psychosis and healthy controls (HC). Mitochondrial complex (I–V) function was measured in monocyte samples (white blood cells) in a multiplex ELISA assay. Complex function is reported as a percentage against each subject’s individual nicotinamide nucleotide transhydrogenase levels (%NNT); a nucleus-encoded protein present in the inner mitochondrial membrane that is closely related to mitochondrial oxidative phosphorylation. A repeated measures ANOVA was performed to test the effect of group (CHR vs healthy controls) on mitochondrial complex (I–V) function. There were no significant differences in mitochondrial complex (I-V) function between CHR and HC (F_(1,40)_ = 0.67, p = 0.42).

**Figure 2:**
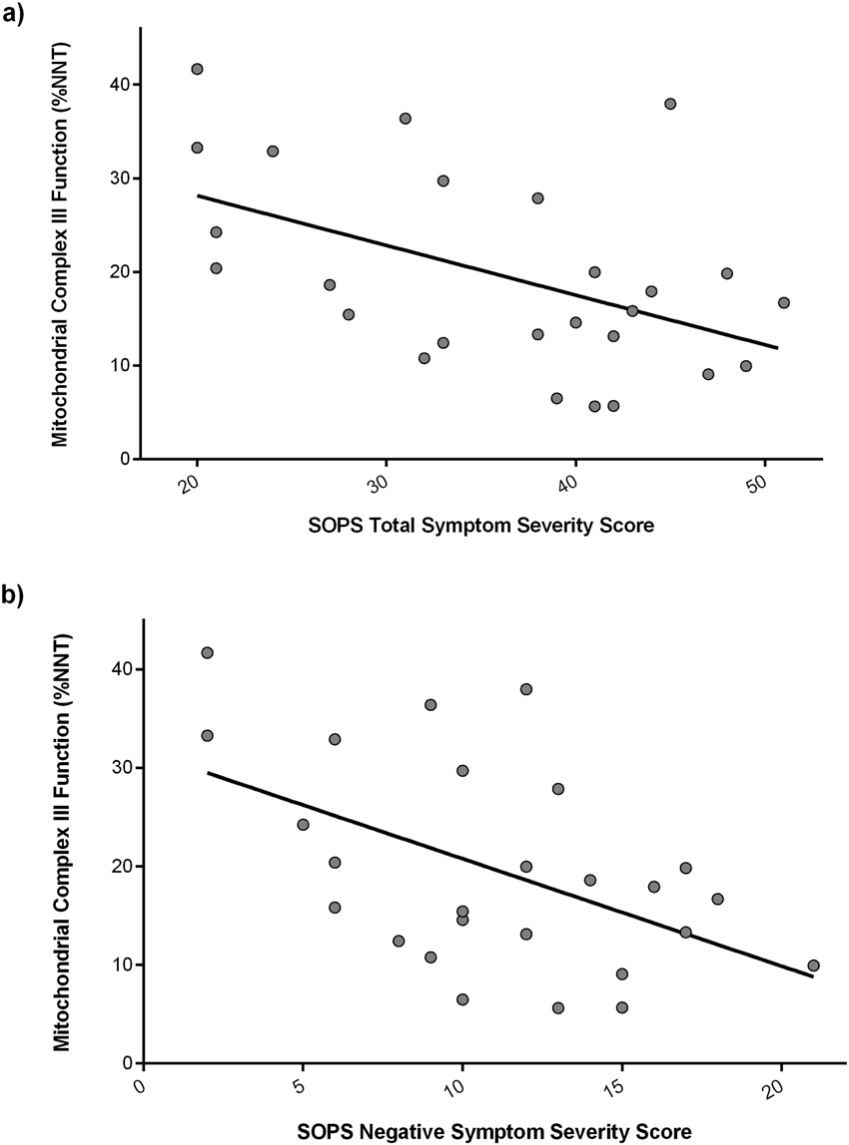
Association between peripheral mitochondrial complex III function and (**a**) total SOPS symptom severity score (r = −0.49, *p* = 0.01) and (**b**) SOPS negative symptom severity score (r = −0.51, *p* = 0.008) in clinical high risk (CHR). Lactate levels were measured in plasma using a colormetric L-Lactate Assay Kit, and are reported in nmol/µL. Bivariate correlations were used to investigate the associations between mitochondrial complex function and symptom severity.

There was no significant differences in mitochondrial complex (I–V) function between CHR and healthy controls (F_(1,40)_ = 0.67, p = 0.42) (Fig. 1). Similar results were also obtained after controlling for tobacco use, or after excluding the CHR individuals currently on antipsychotic medication (n = 5), or positive for cannabis use (n = 2). In addition, there were no significant differences in mitochondrial complex (I–V) function between CHR tobacco smokers (n = 6) and CHR non-smokers (n = 20). Within the CHR group, mitochondrial complex III function was inversely related to SOPS total symptom severity score (r = −0.49, *p* = 0.01; Fig. 2a), which survived correction for multiple comparisons. Follow up analysis revealed a significant contribution of SOPS negative symptom severity score (r = −0.51, *p* = 0.008; Fig. 2b). There were no significant correlations between mitochondrial complex (I-V) function and SOPS positive symptom severity score (*p* > 0.05). In addition, mitochondrial complex V function was inversely related with the RBANS attention subscale in the sample as a whole (n = 42; r = −0.44, *p* = 0.004; Supplementary Fig. S2), suggesting that increased mitochondrial function may be related to poorer cognitive performance. The healthy control group appeared to drive this correlation (r = −0.73, *p* = 0.001; Supplementary Fig. S2), which survived correction for multiple comparisons. There were no other significant correlations between mitochondrial complex function and cognition (*p* > 0.05).

### Peripheral Lactate and Pyruvate levels

**Figure 3:**
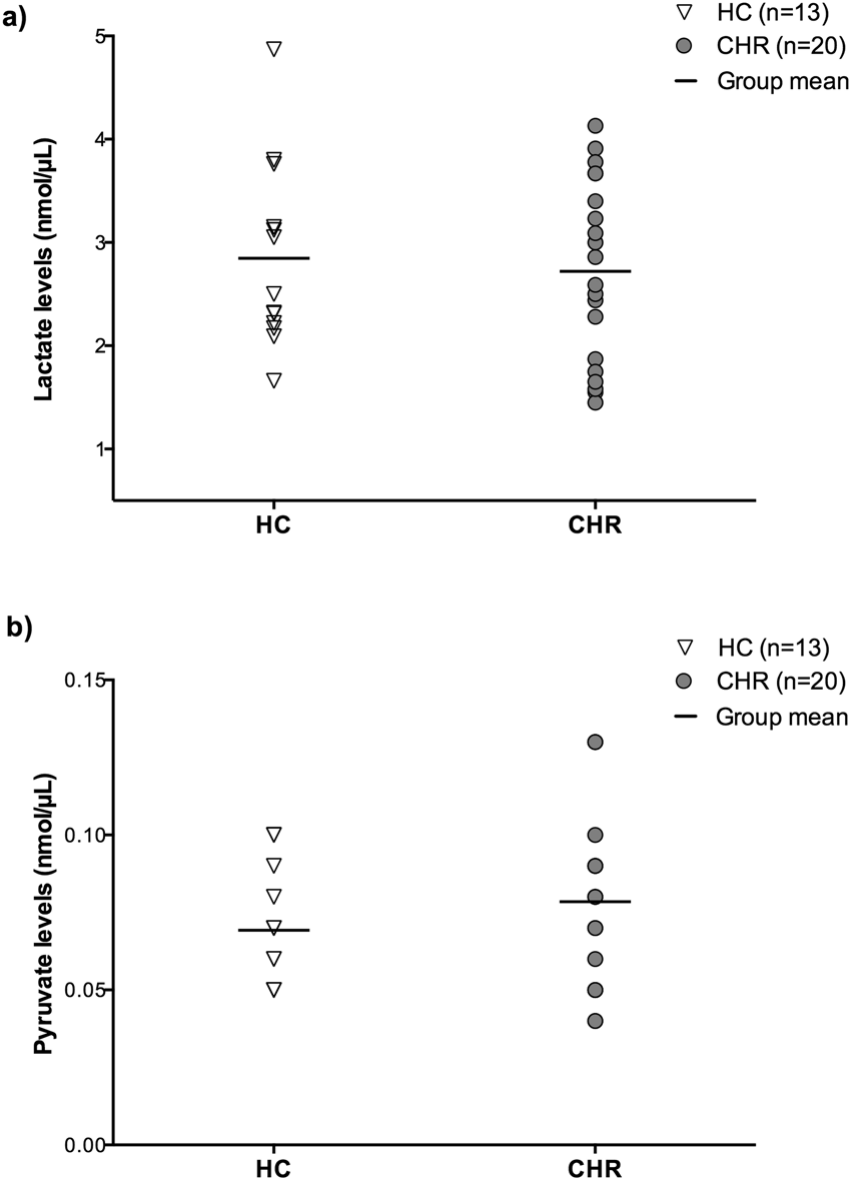
Peripheral lactate (**a**) and pyruvate (**b**) levels in clinical high risk (CHR) and healthy controls (HC). Lactate and pyruvate levels were measured in plasma using a colormetric L-Lactate Assay Kit and a colormetric Pyruvate Assay Kit, respectively, and are reported in nmol/µL. A univariate analysis of variance was performed to test for differences in lactate and pyruvate between groups. There were no significant differences in lactate or pyruvate levels between CHR and HC (lactate: F_(1,31)_ = 0.17, *p* = 0.69; pyruvate: F_(1,31)_ = 1.31, *p* = 0.26).

**Figure 4:**
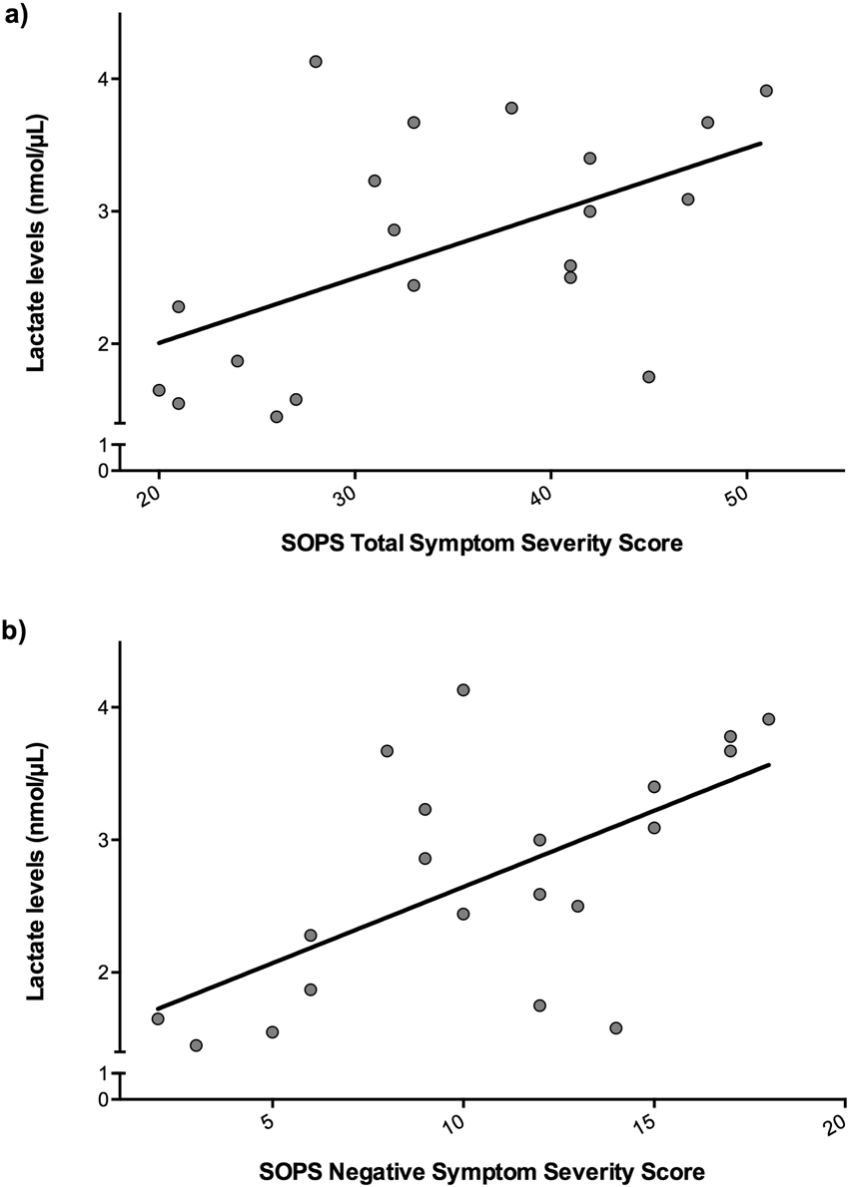
Association between lactate levels and (**a**) total SOPS symptom severity score (r = 0.54, *p* = 0.01) and (**b**) SOPS negative symptom severity score (r = 0.61, *p* = 0.004) in clinical high risk (CHR). Lactate levels were measured in plasma using a colormetric L-Lactate Assay Kit, and are reported in nmol/µL. Bivariate correlations were used to investigate the associations between lactate levels and symptom severity.

There were no significant differences in peripheral lactate (F_(1,31)_ = 0.17, *p* = 0.69) or pyruvate (F_(1,31)_ = 1.31, *p* = 0.26) levels between CHR and healthy controls (Fig. 3). Similar results were also obtained after controlling for gender or after excluding the CHR individuals currently on antipsychotic medication (n = 1), or positive for cannabis use (n = 2). However, in CHR, there was a significant effect of tobacco use on lactate and pyruvate levels (lactate: F_(1,18)_ = 5.18, *p* = 0.04; pyruvate: F_(1,18)_ = 5.87, *p* = 0.03), such that CHR individuals who smoked tobacco (n = 6) had significantly lower pyruvate (53.78%) and lactate (42.06%) levels compared to those who did not smoke (n = 14) (Supplementary Fig. S3). Within the CHR group, lactate levels were positively correlated with SOPS total symptom severity score (r = 0.54, *p* = 0.01; Fig. 4a). Follow up analysis revealed a significant contribution from SOPS negative symptom severity score (r = 0.61, *p* = 0.004; Fig. 4b), which survived correction for number of SOPS subscales. There were no significant correlations between lactate and pyruvate levels and SOPS positive symptom severity score (*p* > 0.05). In addition, no significant correlations were found between lactate or pyruvate levels and cognition in the sample as a whole (*p* > 0.05). However, in healthy controls, pyruvate levels were inversely related to RBANS language subscale (r = −0.72, *p* = 0.006; Supplementary Fig. S4), which survived correction for number of RBANS subscales.

### Mitochondrial function and association with brain microglial activation

**Figure 5:**
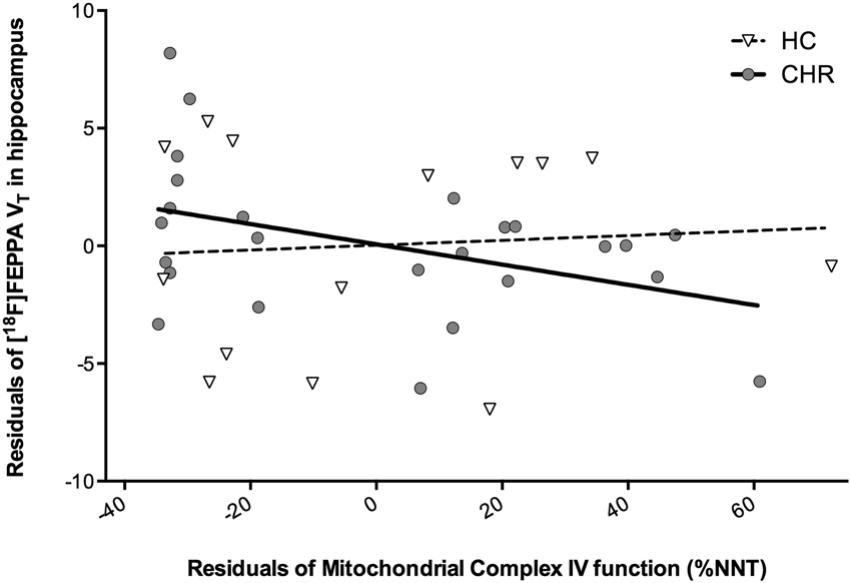
Association between peripheral mitochondrial complex IV function and [^18^F]FEPPA total distribution volume (V_T_) of hippocampus in clinical high risk (CHR) (r = −0.42, *p* = 0.04), but not in healthy controls (HC) (r = 0.07, *p* = 0.82). Mitochondrial complex (I–V) function was measured in monocyte samples (white blood cells) in a multiplex ELISA assay. Complex function is reported as a percentage against each subject’s individual nicotinamide nucleotide transhydrogenase levels (%NNT); a nucleus-encoded protein present in the inner mitochondrial membrane that is closely related to mitochondrial oxidative phosphorylation. [^18^F]FEPPA V_T_ was determined with a 2-tissue compartment model using positron emission tomography (PET). Partial correlations were used to explore the associations between mitochondrial complex function and [^18^F]FEPPA V_T_, controlling for translocator protein 18 kDa (TSPO) rs6971 polymorphism.

There were no significant differences in translocator protein 18 kDa rs6971genotype or PET parameters between groups (Supplementary Table S1). The lack of group differences in [^18^F]FEPPA total distribution volume (V_T_) between CHR and controls have been reported elsewhere^38^. In CHR, there was a significant negative correlation between mitochondrial complex IV function and [^18^F]FEPPA V_T_ in the hippocampus (r = −0.42, *p* = 0.04) (Fig. 5), but not in healthy controls (r = 0.07, *p* = 0.82). This correlation was exploratory and as such was not corrected for multiple comparisons. There were no other associations between DLPFC or hippocampal [^18^F]FEPPA V_T_ and mitochondrial complex function, lactate and pyruvate levels in CHR or healthy controls, or in the sample as a whole (*p* > 0.05).

### Mitochondrial function and association with brain glutathione

There were no significant differences in tissue fraction composition and in FWHM between CHR and healthy controls (Supplementary Table S2). The lack of group differences in glutathione levels between CHR and healthy controls has been reported elsewhere^34^. In addition, there were no significant associations between mPFC glutathione levels and mitochondrial complex function, lactate and pyruvate levels in CHR or healthy controls, or in the sample as a whole (*p* > 0.05).

## Discussion

This is the first *in vivo* study investigating mitochondrial complex function and lactate and pyruvate levels in antipsychotic naïve CHR and its association with microglial activation and glutathione levels. There were no significant differences in peripheral mitochondrial complex function and lactate and pyruvate levels between CHR and healthy controls. In CHR, mitochondrial complex III function was inversely associated with SOPS total and SOPS negative symptom severity score. In addition, lactate levels were positively associated with SOPS total and SOPS negative symptom severity score. Lastly, we report a significant negative association between mitochondrial complex IV function and [^18^F]FEPPA V_T_ in the hippocampus of CHR individuals, that will need to be confirmed in larger studies.

There were no significant differences in mitochondrial complex I–V function between CHR and healthy controls. Our results in CHR are in line with a post-mortem study reporting unaltered complex I function in the prefrontal cortex of schizophrenia patients^14^. In addition, Konradi and colleagues (2004) reported decreased expression of nuclear genes coding for enzymatic complexes in patients with bipolar, but not in schizophrenia^39^. Similarly, a recent ^1^H-MRS study reported no significant differences in glutathione levels, a major antioxidant in the brain, in CHR compared to healthy controls, suggesting unaltered redox status^34^. Moreover, genome-wide association studies have consistently failed to replicate genetic risk factors associated with mitochondrial-related genes in schizophrenia^40^. However, our results are in contrast to several studies reporting alterations, both reductions^17^ and elevations^18,19^ in mitochondrial complex function in peripheral tissues of schizophrenia patients. These conflicting results may be due to differences in cell type, duration of illness, antipsychotic medication, or more critically different clinical stages (CHR vs schizophrenia).

In the CHR group, mitochondrial complex III function was inversely associated with SOPS negative and SOPS total symptom severity score, suggesting that lower mitochondrial function is associated with greater severity of prodromal symptoms. In schizophrenia, two studies have reported a positive association between the observed increase in complex I function and severity of positive symptoms^19,41^; however, consistent with our results, a negative trend level association was reported between complex I function and negative symptoms^19^. In addition, mitochondrial complex V function was inversely related with RBANS attention subscale in the sample as a whole, suggesting that higher mitochondrial function is associated with poorer cognitive performance. Mitochondria have been shown to regulate synaptic transmission, brain function and cognition^3,42^. Thus, changes in mitochondrial function and activity may alter ATP synthesis and consequently brain energy metabolism, which can disrupt normal brain development leading to cognitive impairments often seen in schizophrenia^4,42^. However, future studies are needed to elucidate the role of mitochondria in regulating higher order processes, including cognition.

There were no significant differences in lactate and pyruvate levels between CHR and healthy controls. Our results are in contrast to studies reporting elevated lactate levels in blood cells of schizophrenia patients. However, these studies were mostly conducted in medicated patients. Halim and colleagues^23^ reported a significant effect of antipsychotic treatment on lactate concentrations in post-mortem prefrontal cortex of schizophrenia patients^23^, suggesting that elevated lactate levels might be secondary to antipsychotic treatment rather than related to schizophrenia pathology. In the CHR group, lactate levels were positively associated with SOPS negative and SOPS total symptom severity score, suggesting that higher lactate levels are associated with greater severity of prodromal symptoms. These results are in line with previous studies reporting a positive correlation between elevated lactate levels and neuropsychological symptoms in patients with mitochondrial diseases^43^, including a positive trend level association between *in vivo* brain ^1^H-MRS lactate levels and negative symptoms in schizophrenia^24^. In addition, we observed a significant effect of tobacco use on pyruvate and lactate levels in CHR individuals, such that CHR tobacco smokers (n = 6) had lower lactate and pyruvate levels than CHR non-smokers (n = 14). The interpretation of these results is limited by the small sample size; however, a study reported that during acute abstinence (3–6 hours), as was the case for the CHR individuals in this study, smokers had lower lactate levels compared to non-smokers^44^.

The significant negative correlation between [^18^F]FEPPA V_T_ in the hippocampus and mitochondrial complex IV function is in line with current literature suggesting that microglial activation is associated with mitochondrial dysfunction in the hippocampus. Hippocampal dysfunction has been implicated in the pathophysiology of schizophrenia^45–48^. In addition, imaging studies in patients with schizophrenia show hyperactivity of the hippocampus, thought to be driven by stress-induced loss of inhibitory parvalbumin GABAergic interneurons^45,49^, which may explain the relation between immune activation and mitochondrial function in this brain region. In addition, compromised mitochondrial metabolism often leads to an excess accumulation of ROS that activate redox-sensitive inflammatory pathways^4,27^. Several studies show that mitochondrial ROS modulate innate immunity by activating pattern recognition receptors (PPRs) or by directly activating the inflammasome which triggers a cascade of downstream signaling pathways that activate inflammatory cellular mediators, including glial cells^27^. Activated microglia up-regulate the production of pro-inflammatory mediators and ROS that amplify the oxidative stress response and lead to further mitochondrial damage^4,27,50^. In addition, consistent with our results, a recent study reported that electron transport chain inhibitors stimulated secretion of pro-inflammatory cytokines by activated microglia through enhancing mitochondrial ROS production^51^. However, we did not observe an association between mitochondrial function and glutathione levels in brain, which will need to be confirmed in larger studies.

The results of this study should be interpreted considering the following limitations. First, our relatively small sample size represents a potential limitation. Thus, it is possible that alterations in mitochondrial function in CHR are subtle, and as such require a larger sample size. However, similar sample sizes as in the current study were sufficient to detect a difference in peripheral mitochondrial function between patients with schizophrenia and healthy controls (30 schizophrenia patients, 18 HC)^17^. In addition, exploratory sample size calculations using the present data suggest that to detect group differences (i.e., higher mitochondrial function in healthy controls compared to CHR individuals; the average observed effect size was 0.2), we would need 450 subjects or 255 subjects per group, respectively, for a significance level of 0.05 (two-tailed) with 80% power. Second, there was a significant difference in tobacco use between CHR and healthy controls (*p* = 0.03). Although there was no effect of tobacco use on mitochondrial complex function, CHR tobacco smokers (n = 6) had significantly lower peripheral lactate and pyruvate levels than CHR non-smokers (n = 14); however, the small sample size precludes any meaningful interpretation at this stage. Third, participants were not in fasting prior to blood sample collection, and the effect of this alteration on mitochondrial markers remains unknown. Fourth, we measured mitochondrial function in the periphery and microglial activation and glutathione levels in the brain. Peripheral biomarkers represent whole body alterations, and thus may not reflect alterations in the brain. Further, in our study, mitochondrial complex function was measured in peripheral monocytes which may have a very different metabolic situation compared to brain-specific cells (e.g. neurons, astrocytes). However, parallel alterations in complexes I and V function were found in brain and platelets of patients with Parkinson’s and Alzheimer’s disease, respectively^52,53^, but to date, this is the first study investigating this in CHR. Fifth, although several correlational analyses were performed, each correlation was corrected for number of comparisons or subscales accordingly. Further, based on the calculated sample sizes for the correlational analyses with mitochondrial complex function, we are able to detect a correlation as small as r = 0.52 and r = 0.42 with 80% power within the CHR group (n = 26) and the whole sample (n = 42), respectively. Due to varying sample sizes in the correlational analyses with lactate and pyruvate levels, we are able to detect a correlation as small as r = 0.58 and r = 0.46 with 80% power within the CHR group (n = 20) and the whole sample (n = 33), respectively. All of the above calculations are two-tailed, α = 0.05. As such, the correlational analyses should be interpreted with caution and confirmed in larger studies. Lastly, although the lack of group differences in [^18^F]FEPPA V_T_ and glutathione levels have already been reported elsewhere, the association between mitochondrial function and microglial activation and glutathione levels in brain has never been investigated.

In conclusion, alterations in mitochondrial complex function and lactate and pyruvate levels are not yet evident in the clinical high risk population, but may be involved in prodromal symptom severity, particularly negative symptoms. In addition, we report for the first time, a negative association between mitochondrial complex IV function and microglial activation in the hippocampus in CHR.

## Methods

### Participants

A total of 43 subjects took part in this study, including 27 CHR and 16 healthy controls. Most of the individuals in the CHR group were antipsychotic naïve (N = 22). Individuals in the CHR group were included if they met diagnostic criteria for prodromal risk syndrome as assessed by the Criteria of Prodromal Syndromes (COPS)^54^. CHR individuals were excluded if they had a current axis I disorder as determined with the Structured Clinical Interview^55^ for DSM-IV Axis I disorders (SCID-I). In CHR, clinical status and severity of prodromal symptoms were assessed with the Structured Interview for Psychosis-risk Syndromes (SIPS), the scale of psychosis-risk symptoms (SOPS)^56^. SOPS contains four different subscales of symptom severity: positive symptoms, negative symptoms, general symptoms and disorganization symptoms. Healthy controls were included if they did not have a history of past psychoactive drug use and/or first-degree relatives with a major mental disorder. All participants were excluded if they had (1) clinically significant medical illness, (2) current diagnosis of alcohol and/or substance abuse/dependence, (3) pregnancy or current breastfeeding, and (4) the presence of metal implants precluding an MRI scan. Neurocognitive performance was assessed using the Repeatable Battery for the Assessment of Neuropsychological Status (RBANS)^57^. Urine drug screens were obtained from all participants. This study was approved by the Research Ethics Board at the Centre of Addiction and Mental Health and all participants provided written and informed consent after procedures were explained thoroughly. All procedures were performed in accordance with the guidelines and regulations established by ICH GCP E6 and Health Canada.

### Blood Sampling

Blood samples were acquired from an antecubital vein on the same day as the [^18^F]FEPPA PET scan. Monocytes were extracted from blood to measure mitochondrial complex function. Lactate and pyruvate levels were measured in plasma.

### Mitochondrial Complex Function

Monocyte samples (white blood cells) were used to evaluate complex I-V function in a multiplex ELISA assay. Cells were lysed with phosphatase inhibitors, protease inhibitors and OXPHOX lysis buffer before being centrifuged at 14000 g for 20 minutes at 4 C. 96-well plates were activated with 200 µL of wash buffer for 10 minutes. 25 µL of sample and 25 µL of magnetic beads were added to each well and incubated for 2 hours at room temperature. Plates were washed and 50 µL of detection antibody was added to each well and incubated for an hour at room temperature. Plates were washed and 50 µL of streptavidin-phycoerythrin was added and incubated with samples for 30 minutes. Plates were washed, and 100 µL of drive fluid was added to the wells. The plates were read using Luminex Magpix (EMD Millipore) software, and analysis was performed with xPONENT software. Complex function is reported as a percentage against each subject’s individual nicotinamide nucleotide transhydrogenase levels (%NNT); a nucleus-encoded protein present in the inner mitochondrial membrane that is closely related to mitochondrial oxidative phosphorylation^58^.

### Lactate and pyruvate levels

Peripheral lactate and pyruvate levels were available for the majority of our participants (CHR n = 20, healthy controls n = 13). Lactate and pyruvate levels were measured in plasma using a colormetric L-Lactate Assay Kit and a colormetric Pyruvate Assay Kit, respectively. To quantify lactate, 50 µL of sample was added to 96-well plates in duplicates and incubated with 50 µL of lactate reaction mix (lactate assay buffer, lactate substrate mix and lactate enzyme mix) for 30 minutes at room temperature. The samples were then measured by a microplate reader at 450 nm. To quantify pyruvate, 50 µL of sample was added to 96-well plates in duplicates and incubated with 50 µL of pyruvate reaction mix (pyruvate assay buffer, pyruvate probe and pyruvate enzyme mix) for 30 minutes at room temperature. The samples were then measured by a microplate reader at 570 nm. Lactate and pyruvate levels are reported in nmol/µL.

### PET and structural MRI data acquisition and analysis

PET and MRI data were available for most of our sample (26 CHR and 14 healthy controls). PET data acquisitions have been described in detail elsewhere^59,60^. Briefly, each participant was administered an intravenous bolus injection of 187.72 ± 10.32 MBq of [^18^F]FEPPA for 125 min. All [^18^F]FEPPA scans were performed using a high-resolution CPS-HRRT PET scanner (Siemens Molecular Imaging, Knoxville, TN, USA). Arterial blood was collected for the first 22.5 minutes at a rate of 2.5 mL/min after radioligand injection using an automatic blood sampling system (Model PBS-101, Veenstra Instrument, Joure, Netherland). Manual samples were taken at −5, 2.5, 7, 12, 15, 20, 30, 45, 60, 90, and 120 min relative to time of injection. Dispersion and metabolite-corrected plasma input function was generated as previously described^61^. Proton density (PD)-weighted brain MR images required for the delineation of each region of interest (ROI) were obtained for each subject using a 3 T MR-750 scanner (General Electric Medical Systems). Time-activity curves were extracted for the DLPFC and hippocampus using a validated in-house imaging pipeline ROMI^62^. Total distribution volumes (V_T_) in the DLPFC and hippocampus were derived from the time-activity curve and plasma input function using a two-tissue compartment model, which has been validated for [^18^F]FEPPA quantification^61^. All participants were genotyped based on the translocator protein 18 kDa (TSPO) rs6971 polymorphism as high-(C/C), mixed-(C/T), or low affinity (T/T) binders, as described elsewhere^63,64^.

### ^1^H-Magentic Resonance Spectroscopy

*In vivo* ^1^H-MRS levels of glutathione were obtained from a volume of interest (20 × 40 × 30 mm^3^) positioned in the mPFC (Supplementary Fig. S1). Participants were scanned on a 3 T 750 MR scanner (General Electric HealthCare, Wisconsin, US), equipped with an 8-channel head coil. ^1^H-MRS glutathione data acquisition and analysis have been recently described in detail^34^. Data acquisition parameters were as follow: spectral bandwidth = 5 kHz, number of excitations = 528 (512 of water suppressed, and 16 of water unsuppressed), number of data points = 4096. Briefly, a pair of frequency selective inversion RF pulses^65^ were cycled in an interleave manner between the ‘on’ condition at the frequency of glutathione α-cysteinyl resonance (at 4.56ppm) and the ‘off’ condition at 7.5ppm using TE/TR = 68/1500 ms. Prior to subtraction of the ‘on’ from the ‘off’ condition, the raw MRS data sets were combined in the time domain based on coil sensitivity^66^, followed by frequency correction using the unsuppressed water signal. Subtraction of the two resulting spectra results in the glutathione spectrum at 2.9 ppm. XSOS software was used to quantify the area of glutathione resonance by modeling the glutathione peak area as a linear combination of pseudo-Voigt lineshape functions and then fitted in the frequency domain using a highly optimized public-domain Levenberg–Marquardt nonlinear least-squares minimization routine, MPFIT^67^. The glutathione/H_2_O peak area ratio is reported and expressed in ‘institutional units’. Spectra with unsuppressed water resonance frequency width at half maximum intensity (FWHM) greater than 11 Hz or head motion resulting in incomplete subtraction were rejected and removed from further analysis. Tissue voxel composition (i.e. gray matter, white matter and CSF fractions) was determined using Statistical Parametric Mapping version 8 (SPM8) software, as previously described^34^.

### Statistical Analysis

Demographic measures were compared using chi-square tests for categorical variables and independent samples t-tests or Mann-Whitney U tests (if the data was not normally distributed) for continuous variables. A repeated measures ANOVA was performed to test the effect of group (CHR vs healthy controls) on mitochondrial complex (I–V) function. To test for differences in lactate and pyruvate levels between groups, a univariate analysis of variance was performed, with lactate or pyruvate levels as the dependent variable and group (CHR vs. healthy controls) as the independent variable. In addition, we explored the potential effects of confounding factors (i.e. gender, antipsychotic use, or tobacco) on mitochondrial complex function and lactate and pyruvate levels. Bivariate correlations were performed to examine associations between mitochondrial complex function, lactate and pyruvate and their associations with prodromal symptom severity and cognition. Lastly, we explored the association between mitochondrial function and brain glutathione levels or [^18^F]FEPPA V_T_ (controlling for translocator protein 18 kDa rs6971 polymorphism) using bivariate and partial correlations, respectively. All statistical analyses were performed using SPSS (version 22.0; IBM, Armonk, NY, USA), with *p* < 0.05 considered to be significant.

### Data availability

The datasets generated and analyzed during the current study are available from the corresponding author on reasonable request.

## Electronic supplementary material

Supplementary Information
