# Supplementary material for: Mitochondrial function in individuals at clinical high risk for psychosis
Source: Sci Rep. 2018 Apr 18;8:6216. doi: 10.1038/s41598-018-24355-6 (PMC5906614; doi:10.1038/s41598-018-24355-6)

Mitochondrial function in individuals at clinical high risk for psychosis

Tania Da Silva[1†], BSc; Abbie Wu[2†], BSc; Isabelle Laksono, BSc; Ivana Prce[1], BSc; Margaret Maheandiran[1], MSc; Michael Kiang[4], MD, PhD; Ana C. Andreazza[2,3], PhD*; Romina Mizrahi[1, 3, 4, 5], MD, PhD*.

[1]Research Imaging Centre, Centre for Addiction and Mental Health, Toronto, Ontario, Canada

[2]Department of Pharmacology & Toxicology, University of Toronto, Toronto, Ontario, Canada

[3]Institute of Medical Science, University of Toronto, Toronto, Ontario, Canada

[4]Department of Psychiatry, University of Toronto, Toronto, Ontario, Canada

[5]Campbell Family Mental Health Research Institute, Centre for Addiction and Mental Health, Toronto, Ontario, Canada

†Authors with equal contribution.

*Senior authors with equal contribution.

*Corresponding author: Romina Mizrahi, MD, PhD

PET Centre, Research Imaging Centre

Centre for Addiction and Mental Health

250 College Street, Toronto, Ontario, Canada M5T 1R8

Tel: +1 416 535 8501 Ext. 34508 FAX: +1 416 979 4656

Email: romina.mizrahi@camhpet.ca

Ana Cristina Andreazza, Pharm, PhD

Departments of Psychiatry and Pharmacology

University of Toronto

1 King's College Circle, Toronto, Ontario, Canada M5S 1A8

Email: ana.andreazza@utoronto.ca

SUPPLEMENTARY RESULTS

Supplementary Table S1: TSPO genotype and PET parameters in CHR and healthy controls. Data are presented as mean ± standard deviation.

		**HC (N=14)**	**CHR (N=26)**	**Test value**	***p* value**
**TSPO genotype**	**HAB**	10	14	χ^2= 1.17	*p*= 0.28
	**MAB**	4	12		
**PET parameters**	**Specific activity (mCi/µmol)**	1386.36 ± 945.95	1566.70 ± 848.03	t= -0.53	*p*= 0.60
	**Mass injected (µg)**	2.01 ± 1.38	1.73 ± 1.38	t= 0.57	*p*= 0.57
	**Amount injected (mCi)**	5.00 ± 0.31	5.09 ± 0.26	t= -0.98	*p*= 0.34

Abbreviations: CHR, clinical high risk; HC, healthy controls; high affinity binder, HAB; mixed affinity binder, MAB; PET, positron emission tomography; TSPO, translocator protein 18kDa.

Supplementary Table S2: Tissue fraction composition of the MRS voxel and FWHM in CHR and healthy controls. Data are presented as mean ± standard deviation.

		**HC (n=16)**	**CHR (n=27)**	**Test Value**	***p* value**
	**FWHM, Hz**	7.31 ± 0.70	7.48 ± 1.19	t= -0.52	*p*= 0.61
**Tissue Fraction %**	**Gray Matter**	63.44 ± 3.25	63.71 ± 3.81	t= -0.24	*p*= 0.82
	**White Matter**	20.98 ± 2.53	19.58 ± 2.63	t= 1.71	*p*= 0.10
	**CSF**	15.56 ± 3.59	16.69 ± 3.93	t= -0.94	*p*= 0.35

Abbreviations: CHR, Clinical high risk; CSF, cerebrospinal fluid; FWHM, frequency width at half maximum intensity; HC, healthy control.

SUPPLEMENTARY FIGURES

Supplementary Figure S1: Axial, Sagittal and Coronal views of the voxel placement in the medial prefrontal cortex (mPFC).

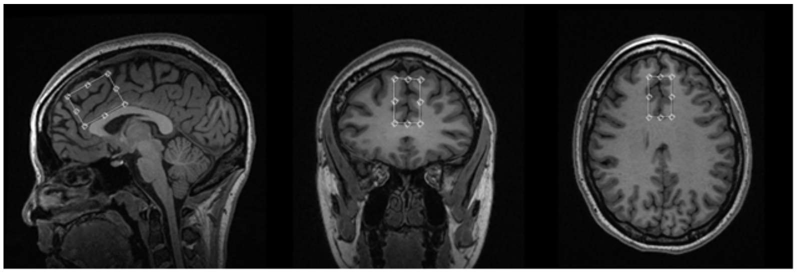

Supplementary Figure S2: Association between mitochondrial complex V activity and RBANS attention subscale in the sample as a whole (r= -0.44, *p*= 0.004) and in healthy controls (r= -0.73, *p*= 0.001). Mitochondrial complex (I-V) function was measured in monocyte samples (white blood cells) in a multiplex ELISA assay. Complex function is reported as a percentage against each subject's individual nicotinamide nucleotide transhydrogenase levels (%NNT); a nucleus-encoded protein present in the inner mitochondrial membrane that is closely related to mitochondrial oxidative phosphorylation. Bivariate correlations were used to investigate associations between mitochondrial complex function and cognition, as measured by the Repeatable Battery of Neuropsychological (RBANS).

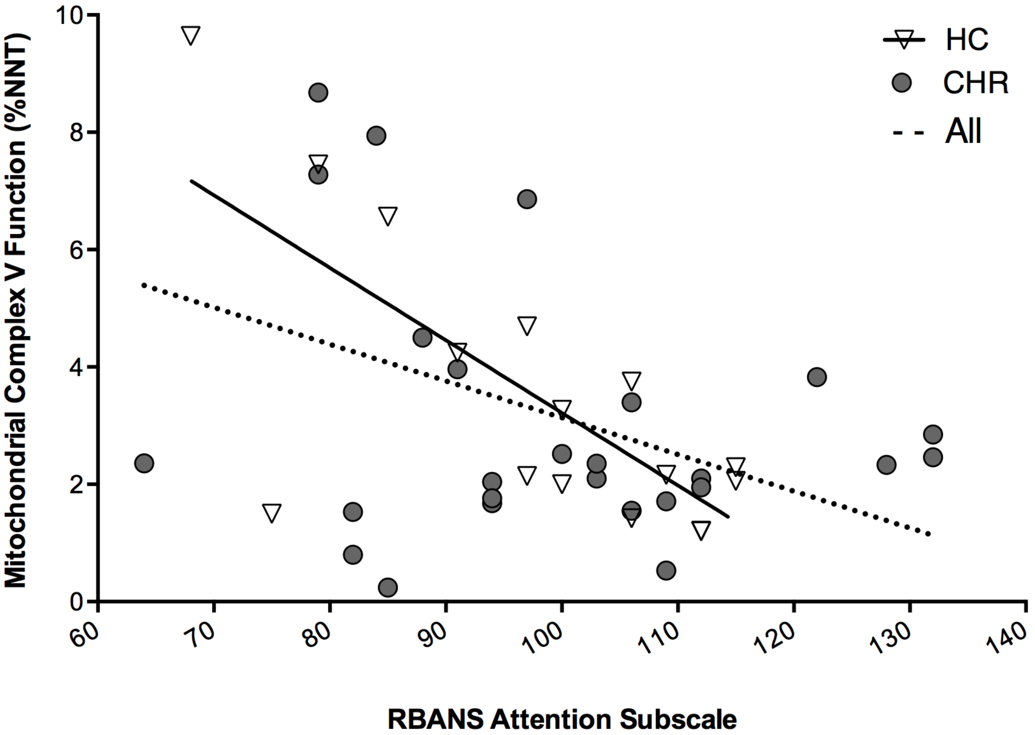

Supplementary Figure S3: Peripheral (A) lactate and (B) pyruvate levels in CHR tobacco users compared to CHR non-users. Lactate and pyruvate levels were measured in plasma using a colormetric L-Lactate Assay Kit and a colormetric Pyruvate Assay Kit, respectively, and are reported in nmol/μL. A univariate analysis of variance was performed to test for differences in lactate and pyruvate between CHR tobacco smokers and CHR non-tobacco smokers. In CHR, there was a significant effect of tobacco use on lactate and pyruvate levels (lactate: $F_{(1,18)}$= 5.18, *p*= 0.04; pyruvate: $F_{(1,18)}$= 5.87, *p*= 0.03), such that CHR individuals who smoked tobacco (n=6) had significantly lower pyruvate (53.78%) and lactate (42.06%) levels compared to those who did not smoke (n=14).

(A)

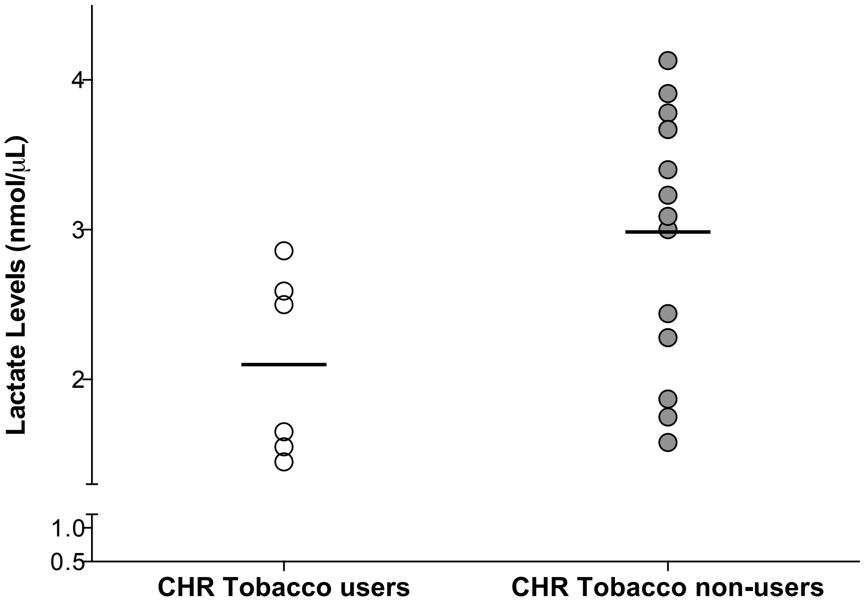

(B)

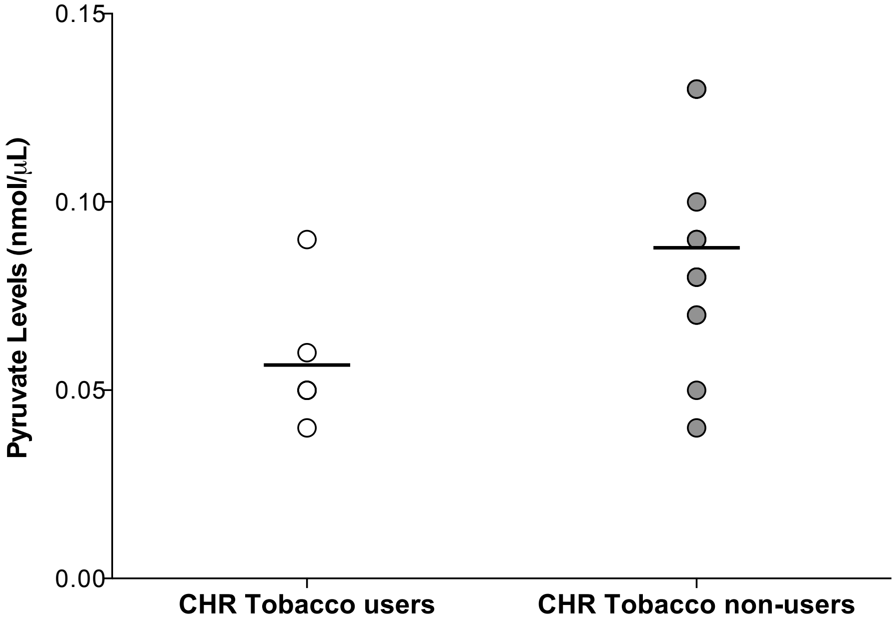

Supplementary Figure S4: Association between pyruvate levels and RBANS language subscale in healthy controls (r= -0.72, *p*= 0.006). Pyruvate levels were measured in plasma using a colormetric Pyruvate Assay Kit, and are reported in nmol/μL. Bivariate correlations were used to investigate associations between pyruvate levels and cognition, as measured by the Repeatable Battery of Neuropsychological (RBANS).

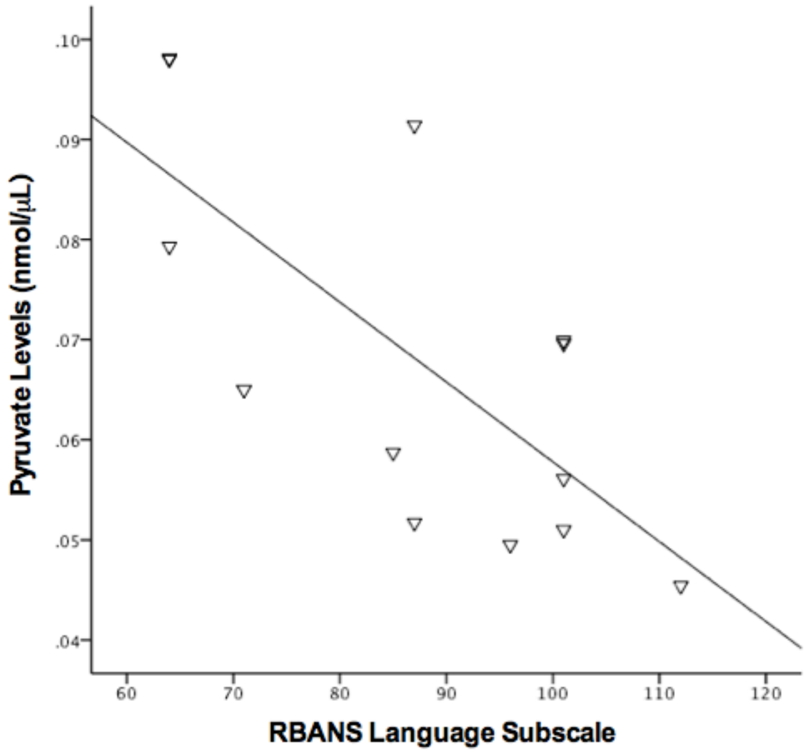

Supplement: Supplementary file 1 — Supplementary Information [file 41598_2018_24355_MOESM1_ESM.pdf]
